# Alpha-synuclein research: defining strategic moves in the battle against Parkinson’s disease

**DOI:** 10.1038/s41531-021-00203-9

**Published:** 2021-07-26

**Authors:** Luis M. A. Oliveira, Thomas Gasser, Robert Edwards, Markus Zweckstetter, Ronald Melki, Leonidas Stefanis, Hilal A. Lashuel, David Sulzer, Kostas Vekrellis, Glenda M. Halliday, Julianna J. Tomlinson, Michael Schlossmacher, Poul Henning Jensen, Julia Schulze-Hentrich, Olaf Riess, Warren D. Hirst, Omar El-Agnaf, Brit Mollenhauer, Peter Lansbury, Tiago F. Outeiro

**Affiliations:** 1 The Michael J. Fox Foundation for Parkinson’s Research, New York, NY USA; 2 Department of Neurodegenerative Diseases, Hertie-Institute for Clinical Brain Research, University of Tübingen, Tübingen, Germany; 3 German Center for Neurodegenerative Diseases (DZNE), Tübingen, Germany; 4 Departments of Neurology and Physiology, UCSF School of Medicine, San Francisco, CA USA; 5 German Center for Neurodegenerative Diseases (DZNE), Göttingen, Germany; 6 Department for NMR-based Structural Biology, Max Planck Institute for Biophysical Chemistry, Göttingen, Germany; 7 Institut François Jacob, MIRCen, CEA and Laboratory of Neurodegenerative Diseases, CNRS, Fontenay-aux-Roses, France; 8 Biomedical Research Foundation of the Academy of Athens, Athens, Greece; 9 First Department of Neurology, Medical School of the National and Kapodistrian University of Athens, Athens, Greece; 10 Laboratory of Molecular and Chemical Biology of Neurodegeneration, Brain Mind Institute, Faculty of Life Sciences, EPFL, Lausanne, Switzerland; 11 Department of Psychiatry, Neurology, Molecular Pharmacology and Therapeutics, Columbia University, New York, NY USA; 12 Division of Molecular Therapeutics, New York State Psychiatric Institute, New York, NY USA; 13 University of Sydney, Brain and Mind Centre and Faculty of Medicine and Health, School of Medical Sciences, Sydney, NSW Australia; 14 Neuroscience Program, The Ottawa Hospital, Ottawa, ON Canada; 15 University of Ottawa Brain and Mind Research Institute, Ottawa, ON Canada; 16 Division of Neurology, The Ottawa Hospital, Ottawa, ON Canada; 17 Aarhus University, Department of Biomedicine & DANDRITE, Danish Research Institute of Translational Neuroscience, Aarhus, Denmark; 18 Institute of Medical Genetics and Applied Genomics, University of Tübingen, Tübingen, Germany; 19 Neurodegenerative Diseases Research Unit, Biogen, Cambridge, MA USA; 20 Neurological Disorder Research Center, Qatar Biomedical Research Institute (QBRI), Hamad Bin Khalifa University (HBKU), Qatar Foundation, Doha, Qatar; 21 Department of Neurology, University Medical Center Göttingen, Göttingen, Germany; 22 Paracelsus-Elena-Klinik, Kassel, Germany; 23 Lysosomal Therapeutics Inc, Cambridge, MA USA; 24 Department of Experimental Neurodegeneration, Center for Biostructural Imaging of Neurodegeneration, University Medical Center Göttingen, Göttingen, Germany; 25 Max Planck Institute for Experimental Medicine, Göttingen, Germany; 26 Translational and Clinical Research Institute, Faculty of Medical Sciences, Newcastle University, Newcastle, UK

**Keywords:** Parkinson's disease, Parkinson's disease

## Abstract

With the advent of the genetic era in Parkinson’s disease (PD) research in 1997, α-synuclein was identified as an important player in a complex neurodegenerative disease that affects >10 million people worldwide. PD has been estimated to have an economic impact of $51.9 billion in the US alone. Since the initial association with PD, hundreds of researchers have contributed to elucidating the functions of α-synuclein in normal and pathological states, and these remain critical areas for continued research. With this position paper the authors strive to achieve two goals: first, to succinctly summarize the critical features that define α-synuclein’s varied roles, as they are known today; and second, to identify the most pressing knowledge gaps and delineate a multipronged strategy for future research with the goal of enabling therapies to stop or slow disease progression in PD.

## Genetics of synucleinopathies

### What we know

The first pathogenic mutation in the *SNCA* gene, encoding for α-synuclein (aSyn) was discovered in a large family from Southern Italy, the “Contursi kindred” in 1997 and in three smaller families of Greek descent^1^. The same mutation was later found in other cases of familial Parkinson’s disease (PD), mostly in patients of Greek descent, indicating the presence of a founder effect^2,3^. Independent A53T mutations have been identified in Korean^4^ and Swedish^5^ patients. Seven other mutations in the *SNCA* gene have been described for which co-segregation in autosomal-dominant families supports pathogenicity—A30P^6^, E46K^7^, G51D^8^, H50Q^9^, A53V^10^, A53E^11^, and A30G^12^. Today it is undisputed that at least some of these point mutations cause PD with high penetrance, even though the total number of mutation carriers is rather low and not in all of the genetic evidence of co-segregation is unequivocal. Most *SNCA* point mutation carriers have a relatively early onset of disease, in their late 30s or 40s, a rapid progression, and significant cognitive impairment.

In 2003, a large triplication of the *SNCA* locus co-segregating with the disease was identified in a family with autosomal-dominant early-onset parkinsonism and rapid cognitive decline^13^, further strengthening the strong link between aSyn and PD. Duplications have also been found to cause PD, usually with later onset^14^, creating an “allelic row”^15^ that links expression levels of aSyn with its pathogenic effect. While these findings unequivocally establish a role for alterations of coding variants and gene dosage mutations in the *SNCA* gene in the development of PD, they account only for a very small proportion of cases.

Early candidate gene studies have suggested an association of common variability in the *SNCA* locus with PD risk^16,17^, but due to the limitations of this study format those findings were contested. The first well-powered genome-wide association study, however, clearly confirmed this association^18^, and this has been corroborated and extended by multiple studies later^19,20^. Fine mapping of the locus indicates the presence of at least three different signals^21^. Variability in the 3’-region of the *SNCA* gene is linked to PD risk, while a second signal in the 5’ promotor region is stronger in patients with dementia with Lewy bodies (DLB)^22,23^. The *SNCA* locus shows the strongest signal in GWAS of PD and has also been found as a modifier of age at onset of sporadic PD^24^.

Mutations in several genes have been found to cause nigral degeneration and parkinsonism with autosomal-dominant, autosomal-recessive and X-linked inheritance. Not all are consistently linked to aSyn pathology (typically identified as pS129-ASYN-positive inclusions), as recently reviewed^25^. Although still based on relatively small numbers of autopsies, most cases of early-onset recessive parkinsonism caused by mutations in the parkin gene (*PRKN*) exhibit severe and relatively selective nigral degeneration without aSyn pathology while, curiously, a single case of recessive parkinsonism caused by compound heterozygous PINK1 mutations, which are thought to act in the same pathway as parkin, had extensive aSyn-positive Lewy pathology^26^. Autopsies of cases with LRRK2 mutations show variable pathology, even within individual families^27^, including aSyn-positive Lewy pathology, nigral degeneration without clear pathologic aggregates, and tau pathology. Overall, about half of the patients with any LRRK2 mutation and two thirds of those carrying the common G2019S mutation have LB pathology^28,29^. Patients with mutations in the GBA-gene, considered to be the most common strong genetic risk factor for PD, have usually extensive Lewy pathology in a distribution consistent with Lewy body dementia^30^.

Multiple systems atrophy (MSA) is a well-described but poorly understood sporadic synucleinopathy, characterized by severe autonomic dysfunction with parkinsonism (MSA-P) or cerebellar dysfunction (MSA-C). The pathologic hallmark is the presence of glial cytoplasmic inclusions (GCIs) immunopositive for aSyn. Patients with the *SNCA* point mutations A53T and G51D^31^ as well as patients with *SNCA* locus triplication^15^ share clinical and pathologic characteristics with MSA and DLB. While one study suggested association with common SNPs at the *SNCA* locus^32^, this finding could not be replicated in a recent GWAS for MSA^33^ and was probably due to population admixture.

### Gaps challenges and opportunities

While the role of rare coding mutations as well as that of common non-coding variants of the *SNCA* locus have been firmly established as determinants of PD risk, their mechanism of action is still quite unclear. More sophisticated models, such as standardized and automated high-throughput cell culture systems, will have to be developed to enable the reliable study of subtle regulatory effects of risk variants. In the future, elucidation of these mechanisms might help to translate genetic findings, for example by patient stratification, into effective disease-modifying treatments. For the same reason, more needs to be learned about the interaction of genetic variants in other genes with aSyn (epistasis), as well as about the epigenetic regulation of the *SNCA* locus, e.g., by DNA methylation, which might play important roles in mediating the effect of environmental factors on disease risk and evolution. Even the largest existing GWAS data sets still lack power to detect epistatic effects at the genome-wide level, so increasing cohort sizes is still important. The analysis of very large multimodal data sets from patient cohorts and model systems together with in-depth phenotyping and exposure assessment could, eventually, disentangle these complex regulatory networks. The computational tools using artificial intelligence/machine learning (AI/ML) strategies are rapidly being developed, so progress is to be expected in coming years.

## Function and localization of alpha-synuclein

### What we know

Mutations in aSyn can cause a disease that looks like idiopathic PD. Thus, aSyn belongs to a growing set of proteins (LRRK2, β-glucocerebrosidase [aka acid β-glucosidase, d-glucosyl-*N*-acylsphingosine glucohydrolase, or GCase], and others) that contribute to this degenerative phenotype. However, the absence of inherited aSyn mutations in idiopathic PD also means that this disorder arises through a different mechanism. Rather than caused by a point mutation, idiopathic PD must arise through some acquired disturbance in wild-type aSyn that reflects a change in its regulation, behavior, interactions, or effect. This change must be the first step toward degeneration, and we will understand it only by characterizing the function of aSyn. We can describe regulated expression of the *SNCA* gene, post-translational modifications such as phosphorylation, and interactions with other proteins, but it will be difficult to interpret any change as pathologic unless we know what aSyn does in its normal biological context. This is still an unresolved topic, as aSyn can be detected in different subcellular compartments, including the nucleus^34^. Nevertheless, aSyn is enriched in the nerve terminal^35,36^, so this is a site of relevance in the context of its normal function. Understanding the function of aSyn will suggest sites more amenable to therapeutic intervention than multiple processes that lie downstream.

Considering its degree of conservation among vertebrates, loss of all three synuclein genes has remarkably little effect on the survival or function of mice^37,38^. However, it is very clear that overexpression of aSyn (or bSYN) inhibits synaptic vesicle exocytosis^39,40^ and modulates dopamine release depending on specific patterns of neuronal activity^41^. This occurs in the absence of overt toxicity or aggregation but may, nonetheless, involve some form of injury because loss of the endogenous proteins does not substantially alter neurotransmitter release. On the other hand, the synucleins are, predominantly, presynaptic proteins and interact with membranes in vitro. Work from multiple laboratories has also shown that aSyn can tubulate artificial membranes^42,43^. This observation suggests a role in endocytosis, such as the retrieval of synaptic vesicle membrane after exocytosis required to regenerate synaptic vesicles^44^.

aSyn acts specifically on the fusion pore formed when neurosecretory vesicles fuse with the plasma membrane, promoting pore dilation, and accelerating vesicle collapse into the plasma membrane^45^. A defect in pore dilation is observed in neurons lacking all three synuclein proteins, while overexpression promotes pore dilation, suggesting a possible endogenous function. Since the fusion pore does not generally limit the release of small molecules such as glutamate and GABA, this presumably explains the lack of observed effect on neurotransmission. On the other hand, this role for aSyn predicts a dramatic presynaptic defect, particularly after high-frequency stimulation. How can we reconcile this cellular phenotype with relatively intact neurotransmission and behavior? And in the absence of a defect in neurotransmitter release in knockout mice, what is the role of SNARE complex chaperone proposed for aSyn^38^? What does aSyn do in other cells, such as erythrocytes and platelets? What does aSyn do in the nucleus and in mitochondria, even if present at low levels in these compartments? These are open questions that need to be addressed.

### Gaps challenges and opportunities

Since aSyn knockout mice display minor behavioral phenotypes, we hypothesize that aSyn normally serves to maintain presynaptic function and becomes particularly important under certain conditions, such as stress or aging. To test this, we would need a robust assay for the function of aSyn. Ideally, this would be in neurons, and should involve live imaging by light microscopy, with corroboration by electron microscopy. In neurons, such an assay would enable us to determine the effect of mutations associated with PD, assess the physiological relevance of interacting proteins, and determine the potential of aSyn for regulation. Upon establishing such an assay in neurons, it should then be possible, if not preferable, to develop a simpler system for higher throughput analysis of multiple mutations. We would also need an assay to study the behavior of aSyn in this process—when it arrives at an individual exocytic event, how it changes conformation, and when it departs. The information about timing and localization would constrain the mechanisms of aSyn action and suggest how dysregulation might result in disease. Ideally, it would be important to develop an in vitro assay that explores the mechanism of aSyn activity in biophysical detail. One or more of these systems could then be used to screen for compounds that reverse the effect of disease mutations. More important, however, the results would suggest physiological mechanisms that have the same effect, identifying multiple, biologically relevant sites for therapeutic intervention. This holistic approach to the normal function of aSyn should thus provide the understanding needed for disease prevention.

## Abnormal localization of alpha-synuclein in synucleinopathies

### What we know

As described above, under normal conditions, aSyn localizes mainly to presynaptic terminals of neurons. Consequently, aSyn immunostaining in the normal adult brain is seen as light dot-like neuropil labeling, reflecting such presynaptic localization. In the context of certain synucleinopathies, aSyn accumulates within the neuronal soma, in the form of LB inclusions, but also in more diffuse structures, commonly termed “pale bodies”. Furthermore, it is detected in linear aggregated structures termed Lewy neurites, within the neuritic extensions. A recent study combined high-resolution light and electron microscopy with biochemical techniques to characterize the composition of such structures^46^. The authors concluded that such structures contained, at least in some cases, non-fibrillar aSyn, and that the main components of the inclusions were lipids and membranous organelles, including autophagosomes. Such findings, although not definitive due to the nature of the techniques employed that may have missed some fibrillar conformations, point to the possibility that aSyn fibrils may not be the determining building block of these inclusions. In any case, the aberrant localization of aSyn to sites proximal to the synapse suggests that impaired axonal transport may be at play. Alternatively, excess synaptic aberrant aSyn may be transferred towards the cell body for its more efficient degradation, but this process is nevertheless inefficient at best, leading to aSyn accumulation at such proximal sites.

Antibodies against aSyn phosphorylated at S129 classically label prominently aSyn aggregates formed within the cytoplasm and the neuritic extensions in synucleinopathies. Techniques such as proximity ligation assay (PLA)^47^ or the paraffin-embedded tissue (PET) blot in combination with the Protein Aggregate Filtration (PAF) Assay^48^ have been employed to specifically detect oligomeric aggregated species of aSyn, and have shown abundant presynaptic localization, that is not readily appreciated with conventional immunohistochemistry with antibodies against aSyn. Such data suggest that in disease states aggregated aSyn species are also formed in presynaptic terminals. In fact, this has been elegantly shown in a transgenic mouse model of TH-driven expression of C-terminally truncated aSyn in the nigrostriatal axis, where aSyn aggregates are formed presynaptically, “clogging” components of the SNARE complex, and leading to impairment of dopamine release^49^.

aSyn has also been postulated to accumulate aberrantly in synucleinopathies in close affinity to neuronal organellar membranes, such as the ER/Golgi, or mitochondria, thus setting the stage for toxic effects on such membrane-associated compartments or for the initial stages of its aggregation. In particular, aSyn localization within the mitochondrial-associated membrane, which links the ER to the mitochondria, could lead to the disruption of calcium signaling^50^. On the other hand, interaction with the mitochondrial outer membrane may lead to mitochondrial fission, and localization within the inner membrane to disruption of mitochondrial complex I activity, while localization within the ER lumen may lead to disruption of ER-Golgi trafficking and ER stress^51^.

A critical site of putative aberrant interactions of aSyn may also be the lysosomal membrane. It is known that aSyn is in part normally degraded within lysosomes through the processes of chaperone-mediated autophagy (CMA) and macroautophagy^52^. Regarding CMA in particular, some mutant forms, such as A53T or A30P, bind tightly to the transmembrane receptor protein Lamp2a, which is the rate-limiting step in the process, and “clog” the pathway, leading to its dysfunction, and to a vicious cycle of accumulation of aSyn and other CMA substrates^53^. Modified forms of aSyn, such as those with dopamine adducts, may behave similarly to the mutants^54^.

Another interesting feature of altered localization of aSyn in the context of PD is the fact that it accumulates in a diffuse pattern within non-reactive protoplasmic astrocytes^55^, while in certain PD cases, in particular in genetic synucleinopathies, there may be aberrant localization of aSyn in aggregated conformations within oligodendroglial cytoplasmic inclusions^56^, which are also the hallmark of MSA.

### Gaps challenges and opportunities

It will be important to better define the steps of progressive aSyn pathology within the nervous system in both models of the disease and the disease itself at the subcellular level. It is of course difficult to reconstruct this evolution solely from neuropathological studies. Nevertheless, an intense effort should be devoted to following up on the recent data suggesting aSyn may not always accumulate in fibrillar forms in LBs, using a combination of immunohistochemical, ultrastructural and biophysical/biochemical techniques, to decipher the exact conformations of aSyn within Lewy Bodies/Neurites and their likely precursors, the Pale Bodies. A panel of aSyn antibodies, as well as techniques such as the PLA, will need to be used, as abundant aggregated C-terminal truncated species may be missed if one relies solely on phospho-S129 or C-terminal aSyn antibodies. Conformation-specific antibodies with high selectivity for aggregated aSyn conformations on tissue sections would be very helpful in this regard. Special attention will need to be devoted by neuropathologists to the presynaptic compartment. Furthermore, the newer aSyn seeding models offer the opportunity to follow closely over time the evolution of aberrant structures, and it will be important to show how these are formed in distinct subcellular compartments, using techniques such as PLA, as well as biophysical methods and immuno-EM, as recently published^57^. The potential “toxic embrace” of aberrant aSyn species with distinct partners within these subcellular compartments will need to be ascertained, and potentially deleterious interactions targeted with molecular tools. It will be important to integrate observations from neuropathological evaluations and experiments in models of the disease to arrive at a synthesis that will potentially not only illuminate the nature of involvement of aSyn in the formation of the neuropathological hallmarks of PD, but also provide insights into potential detrimental vs. protective functions of these processes.

The interaction of aSyn conformations with membranous compartments is likely to be critical, and more attention needs to be paid on how these cellular players “talk” and influence each other following such interactions. Interactions with mitochondrial, ER/Golgi and lysosomal membranes will need to be dynamically monitored, paying attention to alteration in these organellar functions and reciprocal changes in aSyn conformations; this approach may be of particular importance regarding the interaction of aSyn with Lamp2a at the lysosomal membrane. Highly contested issues, such as the potential role of the nuclear localization of aSyn, in physiological or pathological states, will need to be clarified. More attention needs to be paid to the localization of aSyn in non-neuronal cells in the context of PD, in terms of its subcellular localization and impact on glial physiology. This is of course all the more important for relevant MSA models.

## Alpha-synuclein structure and conformations

### What we know

aSyn is a small 140 amino acid residue-long protein that is primarily found in nerve terminals, and in pathological states it accumulates in Lewy bodies (LBs) and Lewy neurites. Traditionally, aSyn is thought to accumulate in fibrillar forms in LBs, and is known to be post-translationally modified^58^. In the test tube, aSyn is highly soluble^59^ due to an overall low content of hydrophobic residues and the repetitive nature of its amino acid sequence, where seven imperfect repeats constitute the N-terminal half of the protein. The imperfect repeats have the ability to bind to membranes, which triggers the folding into an amphipathic helix^60–62^. In solution, on the other hand, aSyn rapidly exchanges between a wide range of conformations^59,63^. Therefore, aSyn is known as “intrinsically disordered” illustrating that, in vitro, it does not fold into a well-defined globular structure. Because of its ability to sample a wide range of conformations, aSyn can interact with diverse biomolecules including enzymes, chaperones, the cytoskeleton, and many more. Often, these interactions are transient and comparably weak such that aSyn can rapidly associate with different binding partners. Upon binding to different cellular components, aSyn changes its structure. One such example is the complex of aSyn with peptidylprolyl isomerase A^64^. In addition, post-translational modifications (PTMs) have the potential to further modulate the structural properties of aSyn alone or in complex with interaction partners.

Despite the tantalizing complexity in physiological aSyn structure and molecular interactions, pathology and structural biology has firmly established that, in PD and other synucleinopathies, aSyn molecules cluster together into oligomeric species of different sizes and shapes. Experiments in vitro and in cells have shown that aSyn oligomers can bind to and might even insert into cellular membranes, thereby causing cellular dysfunction^65,66^. In addition, many of these oligomeric species rapidly transform into megadalton, highly stable assemblies with hydrogen-bonded cross beta-structure that bind to fluorescent dyes known to stain amyloid fibrils. Increasing evidence suggests that amyloid fibrils of aSyn might not all look alike, and may have different conformations in different synucleinopathies and, perhaps, even in different patients who have all been diagnosed with PD^67–70^, thereby contributing to disease heterogeneity.

### Gaps challenges and opportunities

The structure and dynamics of a biomolecule are intimately connected to its function. The unique arrangement of the atoms of a biomolecule in the three-dimensional space, and its exchange between different conformations, determine how it can interact with cellular components and perform its function. In order to understand what triggers the change from physiological to pathological conformations of aSyn, to understand the molecular consequences of PTMs and genetic mutations and, thereby, to gain insight into the basic mechanisms of sporadic and genetic PD and other synucleinopathies, it is important to determine the structure and understand the dynamics of aSyn in different cellular states. Detailed knowledge of the conformations that aSyn can adopt alone or in complexes with binding partners is critical to enable the development of small molecules that may selectively target aSyn in a specific state. For example, drug development strategies could be designed to target either the monomeric disordered form of aSyn in solution^71^, aSyn bound to membranes^72^, oligomeric aSyn^73,74^, aSyn fibrils, aSyn modified by enzymes^64^, or aSyn bound to chaperones^75^.

To develop small molecules, which interact with a specific form of aSyn, important next steps are required. For example, the intrinsically disordered nature of aSyn in solution, which exchanges rapidly between different conformations and does not stably fold into a globular structure^59,63^, presents significant challenges to the design and rational optimization of small molecules against that form of aSyn to prevent its aggregation into oligomers and fibrils. Computational methods could play an important role in this endeavor, but still lack the accuracy and/or speed to apply them to intrinsically disordered proteins.

We also know relatively little about how aSyn is recognized by chaperones, how these interactions are modulated by PTMs, and how we thus can modulate degradation of aSyn. To this end, greater high-resolution structural information of the complexes of aSyn with different components of the chaperone machinery^76,77^ will be required. Targeted protein degradation through the proteasome or autophagy is also tightly connected to improvements in the ability to design small molecules that bind to the intrinsically disordered aSyn molecule in solution.

Liquid–liquid phase separation of intrinsically disordered proteins drives cellular condensation of biomolecules and the formation of membrane-less organelles in cells. Tau, which is found in deposits together with aSyn inclusions in the brain of patients diagnosed with PD with dementia, undergoes liquid–liquid phase separation and potentially comprises an important aspect of tau physiology and pathology^78–80^. Recently, it was also shown that aSyn can undergo liquid–liquid phase separation in solution, particularly at low pH values^81^. However, it is currently unclear if aSyn undergoes liquid–liquid phase separation in cells and neurons and what the role of this separation in the molecular properties of aSyn would be in the context of PD and other synucleinopathies.

Similarly, increasing evidence suggests that there might not be a single structure of aSyn fibrils present in the brain of patients diagnosed with synucleinopathies. The structure of aSyn fibrils could differ between patients with different synucleinopathies such as PD and MSA, and one cannot exclude that different aggregate structures may exist in different brain regions^82,83^. In addition, PD patients for which the disease progresses differently, or which have different genetic backgrounds might develop aSyn aggregates with different conformations, and aggregates heterogeneity can contribute to the morphological diversity of inclusions^84^. Therefore, it will be important to perform patient-specific molecular pathology based on high-resolution structural analysis of aSyn aggregates in different areas of the brain, to understand the origin of these different aggregate structures and how they affect the function of neurons, astrocytes, oligodendrocytes, and microglia.

## Aggregation of normal and abnormal alpha-synuclein strains

### What we know

The process of aSyn aggregation into species with molecular weights ranging from that of a dimer to assemblies made of millions of monomers has been extensively documented. Until recently, the heterogeneity of the resulting particles, as observed on electron micrograph grids^85,86^, was considered unimportant, as the possible pathological association was not well understood. Recently, the polymorphism of the aggregated species has gained interest as it was hypothesized that it may constitute the molecular underpinning for, and thus the connection to distinct synucleinopathies^87^. Evidence supporting this hypothesis comes from (i) in vivo studies where the injection of different aSyn fibrillar polymorphs correlate with the generation of distinct synucleinopathies^67^, and (ii) amplification and fingerprinting of pathogenic aSyn aggregates originating from patients who developed distinct synucleinopathies^68,88^.

aSyn is often referred to as a natively unfolded, or intrinsically disordered protein. However, truly natively unfolded proteins are unlikely to exist within the highly crowded cellular environment, as they will frequently encounter and interact with other proteins or biomolecules^63^. As discussed above, aSyn populates billions of conformations on its own and upon interacting with partner molecules, ranging from sugars, nucleic acids, lipid molecules, and proteins. When assessing the structure of aSyn, whether in a test tube or in cells using techniques such as NMR, the signal measured is that of the ensemble, and because this ensemble is large, aSyn appears to lack a defined structure^89^. Hence, aSyn molecules in different conformational subsets can interact with molecules in the same or different conformations, through complementary hydrogen bonds. This yields large numbers of species, e.g., low molecular weight oligomers, that can grow indefinitely by incorporating aSyn molecules in conformations compatible with those at the extremities of the aggregates.

Until 2018, all we knew was that the NAC region constitutes the core of fibrillar aSyn. The remaining amino acid stretches were considered exposed to the solvent, but we did not know how.

The conformation of aSyn within the high molecular weight assemblies is particularly important as it defines both the lateral and longitudinal surfaces of the aggregates, as well as the core and architecture of the highly organized piles of molecules^90^. Amino acid stretches involved in the hydrogen-bonded amyloid backbone of the aggregates are, by definition, in a water free environment and are not exposed at the surface of the fibrils. Their three-dimensional organization, and the frequency with which the monomeric aSyn fold that establishes the adequate hydrogen bonds with the aggregate extremities is populated, define the growth rate of the aggregates. The latter determines the rate at which aggregated aSyn assemblies amplify within cells. An aggregate that grows fast is hypothesized to (i) escape better the cellular clearance machinery, (ii) induce aSyn loss of function, (iii) accumulate to a higher extent in affected cells and (iv) spread better between cells when compared to a slow-growing aggregate.

Conversely, the amino acid stretches that are not involved within the amyloid skeleton of the aggregates are exposed at the surface of the assemblies. These likely define the ensemble of partners (lipid molecules, nucleic acids, membrane and cytosolic proteins, extracellular matrix components, etc.) the assemblies interact with^91^. Upon prion-like propagation of aggregated aSyn assemblies, the partner roles and abundance within the plasma membranes of distinct neuronal populations are expected not only to define the deleterious properties of aSyn assemblies but also their tropism for different neuronal cells and the brain regions they target^92–95^. In addition, it is also important to consider the interaction of aSyn with lipids and how it affects lipid homeostasis, as this is likely to affect its aggregation^96–98^. The lateral surfaces of aSyn aggregates also determine the propensity of the assemblies to bundle, and this affects the surfaces of the aggregates as well as their size and ability to be taken up after binding to neuronal cell plasma membranes.

### Gaps challenges and opportunities

Targeting the surfaces of aggregated aSyn holds therapeutic and diagnostic potential. The use of Cryo-EM to determine the structure of aSyn in its aggregated form has revolutionized the field. Indeed, the Cryo-EM structures that are now becoming available allow determining how amino acid stretches that are not part of the NAC region are organized, whether their side chains are exposed or not to the solvent and how those residues are piled up^99–102^. This allows design of highly specific ligands that either change the lateral or longitudinal surfaces of the aggregates and interfere with their prion-like spread or growth, respectively. Establishing these structures will enable the design of ligands with potential therapeutic value (e.g., to neutralize the contribution of the prion-like propagation of aSyn assemblies that are thought to underlie disease progression) and that could be used in the development of biomarkers of disease (e.g., allow aSyn imaging by PET). The growing evidence for the existence of multiple polymorphs adds additional challenges but it may also allow designing generic ligands with extremely strong affinity.

Importantly, the structural data^99–103^ raise concerns about the strategies relying on the use of antibodies directed against the aSyn primary structures and suggest we may need to consider alternatives.

In vitro, we have the ability to aggregate aSyn into distinct fibrillar polymorphs, but we now need to increase our efforts and focus on those that occur in the brains of patients. The recent successful amplification of aggregated aSyn from patient brains by templating methods^68,88^, without the use of extraction and purification procedures that may affect the structures of the aggregates, eliminates a major obstacle in the determination of the structures of disease-relevant aSyn aggregates by Cryo-EM.

## Role of posttranslational modifications in alpha-synuclein in health and disease

### What we know

Post-translational modifications (PTMs) modulate protein structure, function, clearance, and localization. Therefore, PTMs could serve as molecular switches for regulating aSyn functions in health and disease^104^. Thus, elucidating which aspects of aSyn functions, cellular properties and pathology are regulated by PTMs and identifying the enzymes that regulate theses PTMs holds tremendous potential for understanding the biology of the protein and its role in PD and synucleinopathies.

Although several aSyn PTMs have been identified, including specific PTMs that seem to correlate with pathology formation, a complete inventory of aSyn PTMs in healthy and in PD brains does not exist. Among the most commonly observed PTMs in human PD brain are N-terminal acetylation, phosphorylation (at S129 and to a lesser extent at S87, Y39, Y125)^56^, Ubiquitination (mostly monoubiquitination, at several N-terminal lysine residues), N- and C-terminal truncations (5–140, 39–140, 65, 66, 67, 70–140, 1–103, 1–114, 1–119, 1–120, 1–122, 1–133, and 1–35)^105,106^, and nitration (at nY39 and non-specific nitration of tyrosine residues, Y125/nY133/nY136). The nature and distribution of aSyn PTMs in other synucleinopathies remain poorly understood. Interestingly, only phosphorylation at S129 has been extensively studied in biological fluids (CSF, plasma, saliva), although nitrated (at Y39) and a truncated form of the protein (undefined sequence) have been detected in the blood. In vitro, in cell cultures, and in animal models, several additional PTMs have been observed and investigated, including glycation^107^, lysine acetylation^108^, SUMoylation^109,110^, and O-Glc-Nacylation^111–113^. However, the lack of antibodies that specifically recognize these PTMs, and their low abundance in the brain have hampered further studies aimed at elucidating their role in PD and other synucleinopathies.

The majority of aSyn PTMs were identified in studies aimed at investigating aSyn pathology in human brains or in mouse models of PD. Therefore, it was initially thought these PTMs are responsible for triggering aSyn aggregation and LB formation. Because of the lack of knowledge about the enzymes that regulate these PTMs or methods that enable their site-specific introduction into aSyn, most early studies relied on using natural mutations to mimic PTMs (e.g., use of phosphomimetics or truncated proteins^114–117^). Recent studies have shown that phosphomimetic and other PTM-mimicking mutations do not reproduce all aspects of bona-fide PTMs^118,119^, or the dynamic nature of PTMs (e.g., ubiquitination and phosphorylation), which plays a central role in how these PTMs exert their effect in vivo. The use of enzymes that induce non-specific or inefficient modification of the protein leads to the generation of mixtures of aSyn species with a variable degree of modification and usually an undetermined amount of each species. This heterogeneity, although it may better reflect what happens in cells, on a practical level makes it difficult to interpret results or compare data across different studies, especially if the levels of the modified proteins are not quantitatively assessed. Therefore, it is not surprising that studies aimed at investigating aSyn PTMs using different approaches (e.g., in vitro studies on S129 phosphorylation) often report different or contradictory findings^120^.

To address these limitations, different protein synthetic/semisynthetic approaches that enable the site-specific introduction of single or multiple PTMs throughout the aSyn sequence have been developed^121–123^. These advances enabled the generation of homogeneously modified forms of aSyn bearing different types of modifications, thus facilitating the systematic assessment of their effects. Interestingly, in vitro studies on the aggregation of these site-specifically modified proteins revealed that most aSyn PTMs either inhibit (e.g., phosphorylation at Y39^124,125^, S87^126^, S129^118,119^, O-Glc-Nacylation^111–113^, non-specific nitration), or do not influence (e.g., nitration at Y39 or Y125) aSyn aggregation in vitro^127^. It is noteworthy that some of the PTMs (e.g., nY39 and nY125) that did not significantly alter the kinetics of aSyn aggregation still resulted in the formation of fibrillar aggregates with distinct morphological and structural properties when compared to the WT protein, suggesting that PTMs could be a key determinant of which type of aSyn fibril strains are formed, as recently confirmed for semisynthetic pY39 aSyn^125^.

Finally, one major limitation of current approaches to investigate aSyn PTMs is that they are mostly based on aSyn overexpression models rather than modulating PTMs of endogenous aSyn. The discovery of enzymes that modify aSyn at specific residues, including phosphorylation, C-terminal cleavage, acetylation, or ubiquitination should facilitate future studies to elucidate the role of these PTMs in regulating pathology formation by endogenous aSyn. However, differences in the efficiency and specificity of these enzymes, combined with the use of different cellular and animal models and possibly off-target effects, could also lead to variable or conflicting findings. Therefore, it is crucial that the specificity and efficiency of the enzymes are always quantitatively assessed. This would improve reproducibility and allow comparison of experiments across different laboratories.

Although unmodified aSyn can form amyloid fibrils that resemble those isolated from the human brain, recent Cryo-EM studies of aSyn fibrils isolated from MSA brains suggest that brain-derived fibrils exhibit distinct structural features and PTM patterns^69^. Whether the PTMs observed in brain-derived fibrils occur before or after aSyn fibril formation in the brain, and whether they alter the pathogenicity of fibrils remains unknown. Recent studies in neuronal models suggest that PTMs, such as ubiquitination and C-terminal truncation, play important roles in regulating the processing and packaging of aSyn fibrils during the formation and maturation of LB-like inclusions^57^.

Although recent studies have shown that ubiquitination and nitration of monomeric aSyn change dramatically the morphology and structure of the fibrils^128^ in a site-specific manner, there are no reports in the literature on how PTMs influence the structural and toxic properties of aSyn oligomers. Therefore, further studies are needed to determine at what stages during aSyn aggregation and LB formation are different PTMs introduced and how they influence these processes and aSyn-induced toxicity.

### Gaps, challenges, and opportunities

Although non-pathological aSyn can be post-translationally modified, for decades, the interest in aSyn PTMs has been driven mainly by studies of aSyn pathology and biochemical and immunohistochemical analyses. Similarly, the focus on specific PTMs, such as phosphorylation at S129 and ubiquitination, has been driven primarily by the development and availability of reliable antibodies. The high abundance of these PTMs in LBs and other pathological aggregates has also led to their use as markers of aSyn pathology formation. Several other aSyn PTMs have also been found in LBs^56,126,129,130^, but they have received much less attention, in part because of the lack of reliable tools (including antibodies) and assays that allow reproducible detection and quantification of these PTMs. Although a few studies have attempted to map the proteome of aSyn pathological inclusions in the brain, only a handful of studies have focused on profiling the aSyn PTM species in these inclusions^131,132^.

Recently, a comparison of aSyn species from cingulate cortex and occipital cortex in PD patients and controls, using mass spectrometry, reported the identification of 20 different modified forms of aSyn, including C-terminal truncations at 103, 119, N-terminal truncations (71–140, 68–140, 66–140, 65–140), as well as aSyn species that are truncated at both termini^105^. These studies establish the diversity of the soluble and insoluble aSyn proteoforms and underscore the importance of taking this diversity into consideration when developing methods to isolate or quantify aSyn in biological samples.

This diversity, combined with the insoluble nature of aSyn aggregates and inclusions, poses major challenges for precise mapping of aSyn PTMs and experimental reproducibility across different laboratories. Therefore, there is an urgent need to develop reproducible protocols for the isolation of aSyn (soluble/insoluble) species from other brain tissues and inclusions. This will pave the way for conducting comprehensive and unbiased studies to map and define the aSyn proteome in different brain regions and peripheral tissues of healthy individuals and patients with different synucleinopathies. These studies will allow us to create an inventory of aSyn species and define which species or specific PTMs correlate with different types of aSyn pathologies (e.g., LBs, LNs, and GCIs) and/or other types of synucleinopathies. However, the simple detection of aSyn PTMs is not sufficient to implicate PTMs as modifiers of aSyn normal function(s) and pathology formation. It is crucial to move from qualitative to quantitative assessment of modified aSyn species. This is important as the levels and stoichiometry of different forms of aSyn influence its aggregation and toxicity. This will require the development of reagents (protein standards), tools (antibodies/nanobodies), and sensitive and quantitative assays that make it possible to detect and accurately quantify different aSyn PTMs reliably. Another aspect that has been neglected and should be taken into consideration is to ensure that future methods and assays will be capable of detecting aSyn bearing multiple PTMs as this is, most likely, what happens in any biological context. Strikingly, many of the existing PTM-directed antibodies may not recognize aSyn bearing multiple PTMs (e.g., pY125/pS129). This, in addition to other confounding factors, could explain the large variations in measurements of total aSyn or pS129 levels across different laboratories. Therefore, the dynamic properties and complexity of PTMs must also be considered in biochemical studies aimed at mapping aSyn PTMs and when developing methods and assays to detect and quantify aSyn species in brain tissues and biological fluids.

Advances in protein chemical synthesis and semi-synthesis have enabled the generation of homogenously modified forms of aSyn, thus paving the way to reconstruct the entire aSyn proteome, including forms of aSyn that bear multiple modifications^122^ and, therefore, facilitate the development of antibodies and other important tools.

The understanding of the biological/pathological effects of PTMs may also inform on possible therapeutic targets that may include the enzymes that regulate certain PTMs. Furthermore, future studies should focus on assessing the potential of targets in multiple disease models, preferably in the absence of aSyn overexpression.

## The alpha-synuclein interactome

### What we know

The amphipathic A2 alpha-helical structure of aSyn suggests that it binds to both lipids and proteins and that, when bound, the cytosolic face would be available to interact with many cellular components. This impression is borne out in many studies in which hundreds of interacting proteins have been reported, and so a major goal is to determine which ones are important for normal and disease functions.

The ability of aSyn to bind to acidic phospholipids in highly curved membranes was demonstrated in classic studies^61^. Subsequent research bears out their suggestion that this binding is central to the regulation of aSyn-mediated synaptic vesicle trafficking and neurotransmitter release, as detailed elsewhere in this article and in a relevant recent review^40^.

Among studies examining interactions with proteins involved in exocytosis, recent reports describe interactions with members of the synapsin family^133^. The synapsins were originally identified as “Protein I”^134^ that demonstrated roles in synaptic vesicle trafficking^135,136^. Of the three mammalian isoforms, synapsin III has been suggested to be particularly important for the regulation of dopamine release^137^, although more work needs to be done to identify the specific roles of these proteins. These effects may be due to synapsin’s propensity to exist in a gel-like liquid phase state that mediates synaptic vesicle clustering^138,139^. Deletion of aSyn was shown to increase synapsin III and drive its redistribution, resulting in increased dopamine neurotransmission^140^. This suggests that an interaction between aSyn and synapsin drives more efficient vesicle clustering and/ or axonal transport, although the specific steps in how this leads to altered dopamine release are unknown. The deletion of synapsin III is reported to decrease aSyn aggregation and nigral damage^141^, and is reported as a component of aSyn fibrils in LBs^133^. A requirement for synapsins and synucleins to interact to modulate exocytosis has been buttressed by microscopy studies of synaptic vesicle fusion^142^.

There are also a series of studies reporting interactions of aSyn with SNARE complex proteins, particularly VAMP (a.k.a. synaptobrevin)^38,143,144^.

Other types of interactions may be important for the targeted degradation of synucleins. Interaction with the chaperone hsc70, followed by an interaction with the lysosomal membrane protein LAMP-2a could trigger aSyn-specific degradation by chaperone-mediated autophagy^53^. This degradative process appears to be disrupted by pathogenic aSyn mutations and by interactions of aSyn with oxidized cytosolic dopamine^54^.

The number of additional potential aSyn-binding proteins may truthfully be said to be overwhelming. Monomeric and oligomeric forms of aSyn may bind to small GTPases for internalization and sorting in cells^145,146^ and over 100 synaptosome components, including synapsin I, VAMP-2 and hundreds of additional synaptic proteins^147,148^. Wild-type aSyn binds to tubulin and microtubules and may enable appropriate aSyn folding, a function that could be absent in disease-causing mutants^149^. Oligomers may localize in the ER and activate calcium pumps^150,151^. Using proximity assays, 225 proteins were identified in the immediate vicinity of aSyn in living neurons, with that study concluding that most are involved in endocytosis and mRNA metabolism^152^. In addition, altered associations of aSyn with membranes may influence the normal distribution of aSyn-partner proteins, thereby disturbing normal neuronal function^91,94^.

### Gaps challenges and opportunities

There are multiple challenges foreseen in defining how the synucleins act in the nervous system. First, the synuclein proteins are amphipathic, and bind to both hydrophilic and hydrophobic surfaces. They can bind an enormous number of molecules, most of which are probably irrelevant to either normal function or disease. Given the promiscuity of aSyn binding in experimental assays, it is imperative that the field distinguish whether the interactions occur in living cells or instead result from the disruption of cellular components or overexpression of the components. To demonstrate which interactions are important under specific conditions will require painstaking cell biology and physiology studies.

Second, aSyn binds to acidic phospholipids of highly curved membranes. The early identification of this property has proven fundamental to revealing aspects of its presynaptic function, although many controversies remain, including the complex and transient interactions with lipids and presynaptic scaffolding proteins, SNARE proteins and proteins of the cytoskeleton involved in trafficking and endocytosis. However, methods for characterizing transient lipid binding, including complexes expected to form between lipids and presynaptic proteins involved in synaptic vesicle membrane handling, are significantly more challenging than “fishing” for interacting proteins.

Third, protein overexpression leads to “gains of functions”, in that trafficking and distribution are altered, and additional effects occur that would be absent at normal expression levels. These additional functions could be of fundamental importance to the pathogenesis of PD and other synucleinopathies, as indicated by disease in patients with *SNCA* gene multiplications and from the build-up of aSyn-positive aggregates in these diseases, presumably due to loss of normal degradation and trafficking. While fundamental to understanding disease, the elucidation of normal and diseased functions requires painstaking cell biological and physiological approaches.

Fourth, aSyn can be present in multiple conformations, and these structures are challenging to define in situ. The conformations include not only cytosolic, membrane-bound, multimeric, aggregate and fibrillar forms, but also changes due to amino acid modifications, including mutations and PTMs. The binding, trafficking, and turnover of each structure is likely to be different, and designing analyses in cells and animals is difficult due in large part to the inability to define and control the conformations.

Finally, synucleins are not only highly expressed in the brain, but also highly conserved and present in systems ranging from torpedo fish electric organ, developing axons in bird and mammals, and non-neuronal cells including blood platelets (where it may also regulate secretory vesicle fusion)^153^. The function and binding partners of this protein are likely to be adapted for different functions in different circumstances.

## Biology of alpha-synuclein secretion

### What we know

Extracellular aSyn has important implications in driving of PD pathogenesis, as it has been shown to induce cellular demise as well as impairment of synaptic function. Secreted fibrillar aSyn has been implicated in promoting pathology progression in PD. There is accumulating evidence that misfolded aSyn species can spread between cells in a prion-like manner and seed the aggregation of endogenous protein in recipient cells^154^. The levels of extracellular aSyn depend on both the rate of its release from neuronal cells and the efficiency of its removal through clearance pathways. There is an increasing body of evidence suggesting that such clearance mechanisms may include cellular uptake and degradation, as well as proteolysis by extracellular proteases^155^. In this respect, proteolytic processing of extracellular aSyn emerges as a new important field for active investigation with potential implications for therapy.

Of the human proteases, matrix metallopeptidases (MMPs), plasmin, and kallikrein-6 (KLK6) have been shown to cleave aSyn in vitro and in vivo but the therapeutic utility of their activity on aSyn-induced toxicity is still in debate^156^. We and others have shown that oligomeric aSyn species can also be secreted, at least in part, through exosomes^157,158^. Exosomes have been proposed to mediate the transfer of misfolded aSyn and thus facilitate disease transmission, although the precise pathological mechanism remains elusive. In addition, the role of exosomes in the clearance of pathologic aSyn is unknown.

### Gaps challenges and opportunities

Given the fact that aSyn is expressed in essentially all areas of the mammalian nervous system, we expect different types of pathways and neurons to be involved in the release/secretion of aSyn in vivo. Therefore, it is important to elucidate if this release is regulated through the crosstalk of different neuronal circuits. What form(s) of aSyn do neurons naturally secrete in vivo? Cytoplasmic monomers, membrane-bound oligomers, tetrameric assemblies, or others? And which secreted aSyn species, if any, can form toxic conformers?

There is also mounting evidence on the role of C-terminally truncated aSyn species in intercellular spreading. Whether these species are present in the extracellular space in vivo is currently unknown. To answer such questions and capture such species, microdialysis assays with appropriate probes in conjugation with structurally specific antibodies will likely have to be employed. Ion channels such as potassium (K+) and calcium (Ca2+), are important regulators of aSyn release in the mouse brain^159^. Whether dysfunction of these channels can alter aSyn levels in the interstitial space, thereby contributing to the neurodegeneration in PD is unknown. Furthermore, epidemiological data suggests that L-type calcium channels blockers may protect from PD^160^. Thus, examination of whether the expression levels of various Ca^2+^ channels are altered in different brain areas of aSyn transgenic, knockout, or wild-type mice and whether the aSyn released through such channel-regulated pathways is a transmissible form (e.g., exosomal) will be important. Having in vivo data to suggest that neuronal activity via calcium entry is relevant in propelling neuronal propagation can ultimately lead to new approaches to model and understand propagation.

Investigating the role of free and exosome-associated aSyn forms as paracrine signal molecules in disease transmission is another underexplored topic. Evidence that the aSyn cargo per se in extracellular vesicles seeds the aggregation of endogenous aSyn in recipient neurons or whether PD-linked mutations alter the aSyn exosomal cargo is lacking. To address these issues, we will need to isolate exosomes from different neuronal cell types, and identify the aSyn content of the exosomal cargoes, if any, and correlate it back to their cellular identity. To this end, it is important to develop a panel of surface markers that would allow for the identification of a cell-specific vesicular secretome, and appropriate tools to enrich for populations of exosomes of interest. These tools would allow tracking the alterations of exosome-associated aSyn species and their molecular partners as the disease progresses. The mechanisms controlling the release of aSyn and perhaps distinct exosomal subpopulations are also not well understood. Therefore, it is important to assess how different forms of aSyn are processed and targeted by cells for extracellular vesicle or non-vesicular secretion, and whether this regulation is dependent on the presence of other PD-related proteins, or PTM events.

Understanding the preferential mechanism for recipient cells to internalize aSyn (vesicle-associated aSyn vs. free aSyn) will be important to derive novel therapeutic strategies aimed at blocking aSyn spreading. Pharmacological inhibition of exosomal synthesis may help dissecting such pathways and their role.

What happens to released aSyn is also unknown. It has been recently speculated that a percentage of internalized exosomes could be released intact following fusion with endogenous endosomes, thereby amplifying their transmissibility in a disease state^161^. The multifaceted fate of exosomes urges the need to develop models to track exosomes inside cells. Tetraspanins, which are widely used to mark exosomes, are also expressed in endocytic membranes. Lipophilic dyes which are commonly used to label exosomes due to their sensitiveness and ease of use, can also result in self-aggregation. The chemical conjugation of exosomes with quantum dots could offer an alternative for exosomal labeling and it is worth exploring similar directions^162^.

Synaptic activation stimulates the secretion of aSyn and exosomes^163^. As secretion is an active ongoing process, subsequent changes in the local presynaptic milieu followed by the accumulation of misfolded aSyn may also affect synuclein secretion and further impact the synaptic integrity. Moreover, it has been shown that in the early stages of inclusion formation and upon extracellular treatment with oligomeric aSyn, presynaptic activity is rather increased^164^. While it has been shown that extracellular oligomers impair hippocampal long-term potentiation (LTP), it remains to be tested whether exosomes bearing oligomers confer similar synaptic dysfunction^164–166^. Thus, we need exosome-specific reporter animal and cellular models to dissect vesicular trafficking and exosomal secretion to study their role as vehicles of pathologic cargoes. Along these lines, a mouse model expressing GFP-CD63, thereby displaying tagged vesicles, was developed to study the neuroglia communication mediated by exosomes^167^.

With regards to free extracellular aSyn proteolysis we need to identify new proteases and fully characterize known proteases that prevent the polymerization process/degrade aSyn oligomers to inhibit cell-to-cell propagation and pathology spreading. Naturally, secreted proteases with narrow specificity but selectivity for aSyn should be prioritized. Identifying such promising “synucleinases” clearing wild type but also phosphorylated, lipid-associated and mutant forms of aSyn will require degradomic/proteomic analyses using appropriate human CSF and ISF material. To this end, terminal amine isotopic labeling of substrates (TAILS) could be useful for such protease specificity profiling. A critical way forward in the validation of such “synucleinases”, especially with regards to pathology transmission, would be the crossing of available transgenic aSyn mice with the putative protease knockout or over expressing mice.

In conclusion, an improved understanding of the secretory and extracellular proteolytic pathways of aSyn can provide the basis for new biomarker developments and contribute to the development of neuromodulatory or even causal treatment strategies.

## Spreading of alpha-synuclein pathologies

### What we know

In cohorts of longitudinally followed de novo PD patients who die of intervening illnesses throughout their disease trajectory, Lewy pathologies are found initially in a proportion of neurons (usually a minority) in certain brainstem cell groups (monoaminergic and cholinergic neurons) sparing other brainstem regions^168^. Over time (estimated to occur after an average of 13 years), Lewy pathologies are then found in the forebrain concentrating in limbic brain regions^168^. This time frame is similar to the time it takes for Lewy pathologies to accumulate in fetal neurons transplanted into the forebrain of patients with PD (>10 years before Lewy pathologies occur)^169,170^. The tantalizing concept that aSyn itself is spreading from cell to cell in a pion-like (“prionoid”) manner has emerged from these observations. A less popular interpretation of the irrefutable, human pathology findings is that the disease process itself (via a yet to be identified mechanism), rather than aberrant aSyn species, spreads from neurons to other cells to incite aSyn misprocessing. A combination of both mechanisms may also occur in human PD brains and cannot be discarded at this point.

In DLB, it is difficult to determine the pattern of spreading as Lewy pathologies occur from the outset in many brain regions affected in PD, suggesting a more rapid process of spreading potentially due to coexisting Aβ deposition^171,172^. Many DLB patients have coexisting Alzheimer’s disease pathology. In these patients, the pattern of spread of aSyn pathologies is from the amygdala into the limbic system and then beyond^172,173^.

In MSA, a more variable distribution of pathologies occurs with more brain regions involved^174^, including the white matter, which is largely spared in LB diseases (some accumulation in a few glia very late in disease)^55^. Degeneration of neurons in MSA is also more substantial but regionally highly variable^174,175^.

The variability in the patterns of spreading of aSyn pathologies in the different synucleinopathies is consistent with considerable differences in the cellular interactions as well as the biochemical makeup of misfolded aSyn species (see their polymorphism above) underpinning this phenomenon.

### Gaps challenges and opportunities

A major gap is the lack of aSyn ligand neuroimaging that can confirm the patterns of spreading of aSyn pathologies longitudinally in patients. Of course, this may be complicated by the diversity of molecular types of aSyn involved (see above). Coupled with the relatively low density of aSyn pathologies in Lewy body diseases (particularly in early disease stages) vs. multiple system atrophy (with known, different conformations, see above), aSyn pathologies in Lewy body diseases may be very difficult to image^176^. While there are currently several ligands under development^177^, none have been found to be effective in human trials. Without the ability to detect aSyn pathologies in living individuals, it will not be easy to determine if progression or onset can be deterred by any future disease-modifying treatments. As discussed above, knowledge of the location of pathological species of aSyn and their interactors will be critical to understand the cellular requirements for the initiation of their spreading in the brain. The involvement of all cell types that influence these factors will be important, as the site and cell type involved in the initiation of aSyn pathologies remains to be determined. The recent data from studies of spreading in non-human primates emphasize the involvement of circulating cells rather than the direct transmission of aSyn between neurons^178,179^.

In addition to determining factors involved in spreading the different types of aSyn pathologies, factors involved in how rapidly they spread also need to be determined. The different forms of synucleinopathies have greatly different average disease durations (emphasis on averages) with PD patients surviving decades, while DLB and MSA patients have much shorter survival^180^. Of course, for any individual the rate of progression and survival is highly variable^181^. Identifying and targeting factors impacting on the rate of progression in patients with different types of aSyn pathologies could slow down the spread of pathology. Many studies suggest that cellular factors involved with ageing are important (e.g., aSyn spreading only occurs in aged and not young mice^182^), and this may be due to factors affecting the rapidity of spread of aSyn pathologies. Higher aSyn pathologic loads are found in rapidly progressing DLB^183^, while more concomitant neuropathologies occur in the elderly with more rapidly progressive PD^184^. Determining the factors influencing the rapidity of decline in different patients with similar aSyn strains will be important.

## The interplay between alpha-synuclein and the immune system

### What we know

Besides its role in neural cell health and neurotransmission, aSyn plays a significant role in the immune system, as studied in in vitro, ex vivo and in vivo paradigms^185–187^. Unexpectedly, endogenous, murine aSyn confers survival benefits in all viral encephalitis models studied to date, as induced by host inoculation outside the brain, and it reduces the impact of bacterial sepsis in wild-type mice. Moreover, select cytokine release rates as well as phagocytosis efficiency are altered by elevated human aSyn expression in mice and cultured cells^188^. Several authors have described a low level of *SNCA* gene expression and the presence of aSyn protein in monocytes and lymphocytes under normal conditions^189,190^. Altered *SNCA* gene expression and aberrant aSyn metabolism have been reported downstream of virulent microbe exposure including in the brain, neuronal cultures, and the human gastrointestinal tract^191–196^.

A direct role for aSyn in host defenses, for example against viral and bacterial pathogens, and its regulation downstream of inflammation are two aspects of its interaction with the immune system. These results suggest a conserved role for the protein in such processes, possibly including a signaling function. For example, modified forms of aSyn can also initiate neuroinflammatory responses (e.g., through the activation of microglia). This is widely considered to occur as a consequence of disease processes, such as the release of pathogenic aSyn species from dysfunctional neurons, but also from intact cells^197^. A related and new platform for investigations into mechanisms of neurodegeneration has been generated following the identification of T-cell clones directed at aSyn peptides, as isolated from PD patients early on in the disease process^198,199^, and intriguingly, of T-cell clones that recognize β-synuclein peptides playing a role in neuronal injury models of multiple sclerosis^200^.

In addition to its presence in primary immune cells and thus, the possibility of a direct involvement in host immunity, the relatively high expression level of *SNCA* in developing erythroblasts and megakaryocytes as well its high protein concentration in their mature progeny, i.e., red blood cells and platelets, respectively^201,202^, has raised the question as to its critical functions in hematological cells. These may include aspects of iron homeostasis, lipid composition and curvature of cell membranes, vesicle formation and the release of contents by such vesicles^189^. Each of these important processes could indirectly also affect immune responses.

Of note, aSyn expression in primary cells of hematological origin have also provided a valuable model to study aSyn-modulated biological processes. Examples of this include: (i) to study its endogenous metabolism including the formation of oligomers^203,204^; (ii) to explore the relation between monomeric and multimeric species of aSyn vis a vis lipid binding;^205^ (iii) to probe monomer-to-multimer ratios in peripheral cells as a model platform for the pathogenesis of aSyn-linked disease;^97,206–208^ (iv) to utilize the presence of aSyn proteins in hematological cells, plasma, serum and CSF for the exploration of potential biomarkers in aSyn-related pathological conditions, such as PD, DLB, or MSA^209–212^; and (v) to interrogate aSyn metabolism in peripheral tissues in the context of microbiota on epidermal and epithelial surfaces of mammalian hosts^189,193,213–216^.

As of 2021, the overall relevance of *SNCA* expression in peripheral cells and the impact of any immunomodulatory effects by aSyn protein with respect to the pathogenesis of PD, or DLB and MSA, have not yet been elucidated. While the topic of exploring aSyn in immunological (and hematological) functions represents only a small file within the currently active aSyn research portfolio, the implications for the pathogenesis of these disorders—as well as their potential therapies—may be greater than is presently appreciated.

### Gaps challenges and opportunities

From these collective insights, three avenues of future research activities have become apparent. One, because *SNCA* gene is highly expressed outside the brain including at sites of host-environment interactions (e.g., in the olfactory receptor epithelium; enteric nervous system; autonomic nerve fibers of the skin), the effects of distinct *SNCA* alleles on microbiota composition, on microbial disease susceptibility and the host’s defense against colonizations as well as infections within the naso/oropharyngeal, gastrointestinal, genitourinary systems, and skin surfaces should be further examined, both in rodents and primates.

Two, the function of aSyn proteins in the initiation and regulation of inflammatory responses and, vice versa, of the effects of inflammation on aSyn metabolism should be further studied both within and outside the brain. A possible role for aSyn metabolism (including the generation of immunogenic peptides) may lie not only in dysregulated immune responses following neural cell death disease progression, but importantly, could be linked to disease initiation, as suggested by the early detection of reactive T-cell clones in PD subjects. Defining these roles and associated processes may inform new therapeutic targets for intervention.

Three, any change in relative abundance (and half-life) of post-translationally modified forms of aSyn that occurs during immunological responses should be further studied, including in the context of biomarker studies that relate to the diagnosis of PD, DLB and MSA, as well as during their progression.

As of 2021, clinical trials designed to lower aSyn levels and to alter the levels of higher-order oligomer formation are actively being pursued. Select compounds and biologics have already entered phase-II trials. If aSyn itself plays a protective role in aspects of the host’s immune functions, and possibly, in hematological homeostasis, then markedly decreasing its levels systemically may unintentionally alter the risk to virulent microbial infections as well as to blood disorders during undoubtedly long periods of treatment. These concerns regarding the possibility of adverse events need to be considered in the design, safety monitoring and reporting of outcomes in clinical trials henceforth. On the other hand, levels of distinct species of aSyn in peripheral cells, including those of hematological origin and related to the immune system, may serve as surrogate markers for disease-modifying treatments targeting PD, DLB and MSA.

Last but not least, comprehensive epidemiological studies throughout the human lifespan should match the level of scrutiny usually applied to ongoing genome interrogations. Thus, it would be of interest to collect further evidence of the association of typical, late-onset synucleinopathy disorders, such as PD, with altered incidence rates of communicable, microbial illnesses earlier in life (as part of our exposome), such as hepatitis B and C^217^. In this context, it will also be of great interest to determine, in years to come, whether survivors of COVID19 will have a greater risk of developing typical PD than those without a known exposure to SARS-CoV-2^218^. It is intriguing that one year into the pandemic, a handful of case reports have been published to date that have described cases of parkinsonism in subjects afflicted by COVID19, at least one of which has shown spontaneous improvement^219–221^. Typical late-onset PD with Lewy pathology formation represents a “complex disorder”, whereby collective exposure history, genetic risk and related tissue abnormalities will determine the onset of cellular changes and the ensuing propagation of a pathological state, including altered aSyn metabolism and chronic inflammation; the sum of these changes will be modified by the effects of gender and progression in age^222^.

## Cellular models for studying alpha-synuclein pathologies

### What we know

Cellular models of aSyn pathology are often based on expression or extracellular addition of aSyn and they have recently been reviewed^223,224^.

In most healthy neurons in the brain, aSyn is highly concentrated in nerve terminals despite being translated on ribosomes in the cell body. This demonstrates the efficiency of aSyn axonal transport by mechanisms that are not fully understood. aSyn can also be present in the nucleus of certain neurons and this is most easily detectable using antibodies against the phosphorylated S129 residue^34^. The physiological subcellular localization is changed in pathological states where aSyn inclusions exist in axons and cell bodies, while yet poorly defined oligomeric species may occur in other compartments or organelles as well. When modeling synucleinopathies in cells, it should be kept in mind that specialized compartments like nerve terminals and axons do not exist in most cell models using mitotic cell lines, while even primary cultures of rodent nerve cells and human iPSC-derived neurons may not present distinct pre- and postsynaptic compartments when not fully differentiated and polarized.

aSyn exerts at least some of its functions in the small nerve terminals where it is involved in synaptic vesicle turnover but if and how these processes are involved in initiating and/or sustaining disease is unclear.

The pre-synapse is a special domain with respect to proteostatic mechanisms given functional lysosomes are not present in the nerve terminals or distal axons. Cellular defense mechanisms against pathogenic aSyn at the nerve terminal will likely include (i) a presynaptic chaperone system that unwinds aberrant aSyn states into native conformations, (ii) protein catabolism carried out by proteasomes, which can degrade native aSyn^225–227^, (iii) disposal of protein aggregates by encapsulating them in autophagosomes that are subsequently fused with the lysosomal compartment upon retrograde axonal transport, and (iv) release into the extracellular space, as delineated above.

When proteostasis is perturbed and aSyn starts accumulating, the first “symptom” occurring in a neuron may well be related to neurophysiological functions and be presented as abnormalities in excitability and connectivity^228^.

The axon of mature polarized neurons has a special organization of its microtubule system in which all microtubules point their plus-ends toward the distal pre-synapse^229,230^. This differs from most cells and non-polarized neurons in culture, where microtubules are organized with mixed orientation. The polarized nature of axonal microtubules is required for efficient axonal transport. Without exhibiting this polarization, a cellular model will not reflect aSyn’s environment in the brain where it is highly enriched in presynaptic structures.

Deficiencies in the autophagic-lysosomal system may lead to a local build-up of seeding-competent aSyn species. The significance of this system is underscored by the many genetic risk factors for PD and other synucleinopathies, whose gene products are involved in lysosomal biogenesis and autophagic function. The dysfunction of these gene products seems to be of special relevance for aSyn catabolism^231^. Mechanistic insight into how aSyn is chaperoned and catabolized in nerve terminals and axons is lacking and these processes will be difficult to model in cells not possessing these specialized nerve cell structures.

Recent studies suggest Lewy bodies are actively built by the cell as multi-organellar assemblies, and that such inclusions may be modeled in primary neuronal cultures seeded with preformed a-syn aggregates^57^. However, it should be kept in mind that aSyn-rich inclusions are not required for aSyn aggregates to exert toxicity in cell models^150,232^.

aSyn aggregate-dependent dysfunction has been hypothesized to affect a plethora of functions, including altered vesicle turnover, calcium homeostasis, mitochondrial and endoplasmic reticulum functions, and proteostatic mechanisms. Such dysfunctional states may exist for years and, potentially, start as presynaptic dysfunctions with subsequent loss of synapses. Likely during this period, there is a phase where seeding-competent aSyn species exit from the “sick” cells by mechanisms that have yet to be defined and are taken up by neighboring neurons and glia, where they can template and initiate a new round of cellular insults. This may all occur before the initially affected neuron is dead. Modeling these topologically and temporally separated processes is complex and may not be achievable with one single-cell model. However, cell models are amenable to large screening efforts, and thus useful in the initial steps of drug discovery. Therefore, it will be important to continue developing and characterizing models that replicate as faithfully as possible phenotypes and mechanisms of the dysfunctions that occur in nerve cells and in nervous tissue as recently demonstrated^57,233^.

### Gaps challenges and opportunities

When characterizing cell models for studying aSyn biology/pathobiology it will be important to consider the following:

Does the model reflect an effect of aggregated aSyn stress or merely aSyn overload? Most models express aSyn under the regulation of strong promoters, leading to high aSyn levels in the cell body, and such high protein expression may cause toxicity per se^234^. The method of applying preformed aSyn aggregates to template aggregation of physiological aSyn levels has been used successfully in many labs and circumvents the need for high aSyn expression. The use of aggregate inhibitors to rescue the phenotype can also increase the confidence in the observed phenotypes as being truly aggregate-dependent^150,232^.

Do the aSyn constructs used in the models reflect what happens to native aSyn in the brain? Single amino acid mutations, e.g., A53T or A30P, are sufficient to cause autosomal-dominant forms of PD. This exquisite sensitivity to acquire aberrant functions with even small changes should be kept in mind when evaluating the many models relying on tagging aSyn with reporter proteins that are often larger and compactly folded than the aSyn protein itself. In these situations, it is advisable to make attempts to validate the study’s observations using non-modified species or at least to discuss potential caveats.

What specific aSyn subcellular localization do our cell models reflect? Can we draw conclusions relevant to presynaptic or nuclear events, and is this important for the questions of interest?

In summary, our continued progress will depend on careful consideration of what specific defects and mechanisms in synucleinopathies we attempt to model—and how well they are represented in our cell models.

## Animal models for studying alpha-synuclein function and toxicity

### What we know

Animal models have been used extensively to test the function and toxicity of aSyn in detail as they are relatively fast to generate and easy to alter genetically. From the very early discovery of the protein from the electric organ of the Pacific electric ray *Torpedo californica*^36^, analysis of animal material enabled the study of potential physiological functions of the protein as shown already in the very first studies using *Rattus norvegicus*^235^.

Before the discovery of aggregated aSyn in LBs^236^, animal models of PD were mainly based on toxin-induced impairment of the dopaminergic nigrostriatal pathway, which causes a rapid dopamine depletion that mimics advanced disease stages^237^. However, these models miss key pathological events of PD and, more importantly, do not mimic its progressive nature. Deciphering the role of aSyn in PD has led to the development of animal models mimicking central pathological features of such as aSyn-associated neuronal loss and aSyn aggregation^237^. Such models are referred to as disease gene-based models or etiologic models. Several aSyn transgenic mouse models have been produced and various promoters have been used to drive the expression of the transgene, leading to different results^238^. After first being described in cell culture, the relevance of *SNCA* overexpression and its mutations in the process of protein aggregation was also validated in animal models^239^. Since then, animal models have been used for both deciphering disease processes (such as protein degradation or metabolism) as well as for testing therapeutic strategies.

In addition, animals are used to mimic high levels of aSyn that lead to cell death. Viral vector-mediated transgenesis offers a valid alternative to conventional transgenic animals, as recombinant viruses can (i) be injected specifically into brain structures of interest, (ii) be easily adjusted for expression levels, (iii) be targeted to either neurons or glia, and (iv) start to express their payload at any desired age of the animal^237^.

Finally, animal models focus on studying mechanisms involved in the spread and toxicity of preformed aSyn fibrils. While originally investigated in mouse brain^240^, this phenomenon was also reproduced in multiple other animal models including rats^241^ and non-human primates^242^. In these models, cell-to-cell transmission and propagation of misfolded aSyn, could mirror the spread of human pathology, as deduced from the neuropathological observations. In addition, other approaches to develop animal models, such as injection of brain extracts containing aSyn aggregates (from transgenic mice^243^ or patients with aSyn pathology^244^) into the brain, muscles, peritoneal cavity, or the circulatory system of aSyn-overexpressing or wild-type rodents may also support a prion-like cascade in the development of synucleinopathies^67,245^.

In general, some considerations must be acknowledged when using these models. For example, it remains unclear whether recombinant aSyn PFFs adequately reflect pathological species of aSyn (“strains”) that are present in human diseases, thereby questioning their overall physiological relevance. Along the same line, the use of diverse homogenates and the subsequent inflammatory response they trigger needs to be considered with care. Nevertheless, these models are commonly used, highly reproducible, and thus important for the further exploration of aSyn pathobiology.

### Gaps challenges and opportunities

First, it needs to be clear that models of synucleinopathies are not necessarily models of PD. None of the animal models described above perfectly recapitulates PD neuropathology and replicates the clinical syndrome. Each model mimics certain aspects of aSyn biology and pathology, and the use of each model depends on the question being investigated. Thus, scientists should first know the strengths and weaknesses of each model, before selecting the model(s) most suitable to address the experimental question of interest. One should clearly highlight which disease aspect is recapitulated by the model and limit the conclusions accordingly. As different animal models present different complexities of the central nervous system, we need various models and species to capture diverse aspects of the disease (transgenic/genetic vs. injection model). As PD patients are rather heterogeneous with respect to disease onset, progression, symptoms, and neuropathology, diversifying animal models may help us to restage distinct aspects of PD, and therefore, to develop personalized therapies. Moreover, combining different pathways of pathogenesis (such as by creating animals with more than one genetic or environmental risk) may enhance their PD-type pathologies, and thus approximate the phenotypic expressivity seen in humans.

It needs to be clear what is to be modeled and that models are only approximations of the human condition. While the protein coding sequence of human and rodent *SNCA* is highly conserved, several regulatory regions influencing *SNCA* expression levels and transcript isoforms differ between species^246^. Hence, humanized models are essential as they best recapitulate expression and splicing isoforms of human *SNCA* (and other genes) in rodents. Here, we clearly need to improve our definition of a humanized model differentiating between models with only one human gene such as aSyn and those with multiple human genes covering other PD-related proteins.

A consensus in the field is needed on which phenotypic read-outs should be prioritized when characterizing a model of PD. So far, a strong focus was put on motor features, while non-motor features were largely neglected. Indeed, some transgenic models show numerous early non-motor features of PD with impairments in gastrointestinal function, olfaction, and sleep^237^, which could serve as useful prodromal disease markers. In addition to motor deficits and pathology, these non-motor features should be included in preclinical studies to measure therapeutic efficacy.

For viral vector-based models, a clear drawback is the necessity to stereotaxically inject the vectors into each individual animal. Preps from different sources may vary in terms of protein expression by 10 to 100-fold, even though the vector genome and the vector genome titer are identical. It would be ideal to use just one promoter/AAV serotype to achieve reproducible results. Given that almost all known (patho-)physiological effects of the synucleins are concentration-dependent, i.e., depending on the kinetics and level of expression, the choice of the “latest, most efficient” vector is not necessarily justified, as robust overexpression of synucleins might easily cause supra-physiological effects, which are not relevant for patients. Hence, aSyn expression levels need to be tightly controlled.

In general, there is a critical need for standardizing the tools we use to characterize the animals. Furthermore, consistency in experimental factors is likely to enhance experimental data reproducibility.

With respect to transgenic models, several observations have shown that the phenotype can change over time. Hence, guidelines on good practices to avoid phenotype vanishing and discrepant phenotypes between labs are needed.

The role of the genetic background in a model must be considered when generating a model. Several transgenic mouse lines are based on a background expressing endogenous murine aSyn, thereby complicating the interpretation of findings in these lines.

Another limitation of rodent models for modeling neurodegeneration is their lack of neuromelanin. The pigment neuromelanin accumulates over time in the SNpc dopaminergic neurons of macaques but not in rodents. Non-melanized neurons have been shown to be less vulnerable to neurodegeneration than melanized neurons both in MPTP-treated primates^247,248^ as well as in PD patients^249^. One might argue that, perhaps, only primate dopamine neurons are vulnerable enough to degenerate and, hence, rodent dopamine neurons in general are less suited, because of their higher plasticity, to model human degenerative processes.

A final, more general recommendation applicable to a wide range of research fields is that *negative* results need to be shared and published. To really move the field forward, it is crucial to share results that are unexpected or contradictory to previous studies to enable a proper interpretation of relevant findings.

## Alpha-synuclein antibodies: tools and therapies

### What we know

To date there are no reagents that have surpassed the utility of antibodies in biomedical science. Given their high specificity and affinity to the target antigen(s), antibodies have found their way from a simple detection reagent to some of the most promising immunotherapeutic agents in neurodegenerative diseases.

Several antibodies have been developed targeting different regions as well as various forms of aSyn. Antibodies were generated against purified LBs^250^, its non-amyloid component (NAC), and to N-terminal and the C-terminal regions of the full-length protein^251,252^. Subsequently, in order to further differentiate aSyn from its pathogenic form, attempts were made to generate antibodies against modified forms including oxidized/nitrated forms^253^, various aggregated forms^254,255^ and modified oligomeric forms^256^. More recently, various groups have generated conformation-specific antibodies targeting specifically what are considered the pathogenic forms of α-syn^257–259^.

Given the potential pathological cell-to-cell transmission of aSyn (see sections above) the concept of therapeutically targeting this mechanism with antibodies has been tested preclinically and is being evaluated in ongoing clinical studies. Both active and passive immunotherapeutic approaches have been shown to reduce aSyn pathology in rodent models^260–262^. Clinical trials were initiated in 2014 with PRX002, a humanized version of mouse monoclonal antibody, 9E4^263,264^ and in 2015 with BIIB054, a human derived aSyn antibody^265,266^, both of these antibodies are currently in Phase 2 clinical trials. Table 1 shows the status of all aSyn immunotherapies that we are currently aware of.

**Table 1:** Current status of aSyn immunotherapies.

Asset	Company	Isotype/epitope	Current clinical stage^a^	Posted completion date for current clinical trial^a^
Prasinezumab(PRX002)	Prothena/Roche	Humanized IgG1/115–126	Phase 2	February 2021 (primary) (June 2026: 5-year extension)
Cinpanemab(BIIB054)	Biogen/Neurimmune	Full human IgG1/1–10	Phase 2	Trial discontinued in February 2021
MEDI1341	AstraZeneca/Takeda	Human IgG1 agly (Fc Null)/102–130	Phase 1	January 2021
Lu AF82422	Lundbeck/Genmab	Human IgG1/112–117	Phase 1	December 2020
ABBV-0805	Abbvie/Bioarctic	Humanized (IgG1?)/121–127	Phase 1	Withdrawn (strategic considerations)
AFFITOPE PD03A	Affiris	Synthetically produced α-syn-mimicking peptide used as an active immunotherapy	Phase 1	August 2016

^a^From clintrials.gov on February 2021.

Several questions/challenges that are being faced in the development of these immunotherapies include:

How do we link aSyn pathology and progression of neuropathology to clinical symptoms? Despite a substantial body of evidence linking aSyn to PD, we still do not know when to therapeutically intervene or, more importantly, if/when a “point of no return” cf. aSyn pathology has been reached. Intuitively the earlier the better, but this may require prohibitively long and expensive trials in prodromal disease.

What “species” of aSyn to target? Owing to the complex heterogeneity of aSyn (see above), it is not known what forms of aSyn should be targeted. The antibodies currently in development recognize either the N-terminus of aSyn (BIIB054) or the C-terminus (all other antibodies) see Table 1. In general, they have all been shown (or claimed) to bind aggregated aSyn. However, a direct comparison across these clinical antibodies is lacking and represents an addressable gap for the field. Whether the antibodies target a truly relevant pathological species, which is present extracellularly remains to be determined. The ongoing clinical studies may, somewhat empirically, define this but the number of other variables is extensive and likely confounding.

How do we measure target engagement? There continues to be gaps in reagents/biomarkers to measure the target engagement in the brain or CSF (and how relevant is CSF for such measurements?). There is no aSyn PET ligand available yet, and levels of pathological aSyn (which form?) in CSF are low. While there has been exciting progress in the RT-QuiC/PMCA assays^267,268^, there is still need for further validation and development before these assays can be used in the clinic, even for exploratory endpoints. Until there are better biomarkers we will be limited to correlative clinical endpoints, limiting the ability to optimize dosing levels/frequency.

Can we get sufficient antibody into the brain? One of the biggest tasks in finding a treatment strategy for diseases affecting the brain is overcoming the blood brain barrier (BBB). Given the high molecular weight of antibodies, it is even a greater challenge to make them cross the BBB. To overcome these hurdles, antibodies have been engineered into smaller fragments without hindering their affinity or specificity to the target antigen. Some of these engineered recombinant antibody fragments, including single-chain variable fragments (scFv), diabodies, triabodies, minibodies and single-domain antibodies, are currently being explored for their efficiency compared to the full-length antibodies. Various scFvs were generated using monomeric aSyn^269^, synthetic libraries^270^ and naive human scFv libraries^271^. scFvs specific for pathogenic forms of aSyn, including oligomers, were also generated^272–275^. Single-domain antibodies like nanobodies have also been developed against aSyn^276,277^. Nanobodies were also found to reduce aSyn oligomer-induced cellular toxicity making them potential candidates for immunotherapeutic agents^278^.

Despite these challenges, clinical development continues for at least five aSyn-targeted immunotherapies. We anticipate that the field overall will learn from these studies, and also from the Abeta immunotherapies for AD (http://investors.biogen.com/news-releases/news-release-details/biogen-plans-regulatory-filing-aducanumab-alzheimers-disease), to provide a therapy for PD that addresses the key pathology and has the potential to alter disease progression.

### Gaps challenges and opportunities

Although there have been considerable advances in understanding the structure of aSyn, we still lack thorough knowledge on how aSyn develops into distinct pathological phenotypes in synucleinopathies, and this possesses a great challenge for antibody-mediated immunotherapy. Recently, amplification assays such as PMCA and RT-QuIC, mentioned above, have shown to have potential application in amplifying disease specific strains of aSyn. Validating and improving these assays would aid in the identification, isolation, and clinical characterization of a particular strain of aSyn, potentially allowing for stratification of patients for immunotherapy with strain-specific antibodies.

Identification of aSyn in extracellular biological fluids has helped researchers to better understand the pathogenesis of synucleinopathies. However, it is still unknown what percentage of aSyn is present in the extracellular space and how much is accessible to the antibodies, as α-syn tends to bind other proteins, which may hinder the epitopes being recognized by the antibodies. Hence a clear and better understanding of aSyn in the extracellular form would be beneficial.

The target for immunotherapy using any given antibody should also be properly studied. As aggregation of proteins is observed across a spectrum of neurodegenerative disorders, lessons should be learnt from the recent failures, and one potential success, from the antibody-based immunotherapy trials for AD. The presence of concomitant pathologies in neurodegenerative disorders makes it even more challenging to identify the right target for an efficient therapy. Synucleinopathies are often present with abnormal levels of Aβ or Tau along with aSyn, and targeting not only the pathogenic form of aSyn, but also other coexisting abnormal proteins, should be considered. Therefore, immunotherapy using a cocktail of antibodies may yield a more successful intervention in a complex disease such as PD. Alternatively, bispecific antibodies, targeting two different antigens could be also used as therapeutics agents once the combination of target molecules has been properly identified and validated. Regardless of the developments in disease understanding and treatments, antibodies will continue to be a critical tool and potential therapeutic.

## Alpha-synuclein as a biomarker for PD

### What we know

There has been strong interest in detecting aSyn in extracellular fluids as a biomarker of synuclein aggregation disorders (in analogy of tau protein and β-amyloid in cerebrospinal fluid samples in AD patients corresponding with pathological hallmarks of the brain). The diagnostic accuracy of clinically diagnosed synuclein aggregation disorders has been very poor, especially in the early stages of the disease^279^, which hampers the success of early clinical trials. aSyn and other biomarkers have therefore been investigated as biomarkers for state (for the early diagnosis), but also rate (objective progression markers), fate (prodromal conditions like isolated REM sleep behavior disorder) and trait (e.g., the different phenotypes in PD).

The presence of aSyn in cerebrospinal fluid (CSF) was proven by mass spectrometry and followed by the development and validation of several enzyme linked immunoassays (ELISA) for its quantification^280,281^. CSF samples from several cohorts were analyzed and showed overall a 10-15% reduction in total aSyn levels in aggregation disorders, i.e., PD, MSA, and DLB^282,283^. This decrease was also prevalent in newly diagnosed, de novo PD subjects^284^. These first cross-sectional cohorts were mainly single center and next validated in multicenter settings in the Parkinson’s Progression Marker Initiative (PPMI) cohort^285^. In the following years the recruitment of longitudinal cohorts enabled the analysis of CSF aSyn in longitudinal cohorts like DeNoPa^286^ and PPMI^287,288^, where no clinically meaningful longitudinal change of CSF aSyn was detected. Since the prodromal state of aSyn disorders is well-defined with isolated REM sleep behavior disorder (iRBD), hyposmia and other non-motor symptoms^289^, several cohorts (e.g., PARS^290^, PPMI, DeNoPa) also enabled the recruitment and longitudinal follow-up to study these premotor stages of aSyn disorders and develop biomarkers for these very early stages and possible conversion.

In addition to the results on total aSyn, an assay for oligomeric aSyn showed promising results, that need further and independent validation. aSyn PTMs have been systematically investigated by mass spectrometry (IP-MS/MS) showing that, in CSF, post-translationally modified species of aSyn exist at very low levels, at best. Data from the quantification of aSyn phosphorylated at serine 129 by various ELISA show inconclusive results with either elevated pS129-aSyn or unchanged levels in PD compared to controls^291^. More promising over the last years are the seed aggregation assays (SAAs) with sensitivities and specificities above 80 or even 90%^267,268^ and a high congruency of analyses of the same samples in different laboratories^292^. This antibody-free approach was inspired from the prion field and has been developed by several independent groups. Thereby aggregation-inducing aSyn seeds present in biospecimens are monitored by sequential amplification in vitro and detected with thioflavin-T, resulting in a fluorescent signal that is proportional to the concentration of the aSyn seed in the biospecimens. While the quantitative and high-throughput nature of these assays also in better accessible biological fluids (i.e., blood, saliva) still must be improved, newer analyses even indicate (1) the ability to identify prodromal subjects at risk and (2) seeding dynamic differences in PD and MSA possibly due to different conformational aSyn strains^70,293^.

In addition, studies on aSyn detection in peripheral tissue, such as skin, colon and submandibular gland showed diagnostic potential for PD and for prodromal subjects with iRBD^294–296^. To analyze the intraindividual “levels” and distribution of aSyn in three different fluids (CSF, blood, and saliva) and three different tissues (skin, colon, and submandibular gland), these biosamples were collected in 20 healthy controls and 60 PD subjects cross sectionally in the Systemic Synuclein Sampling Study (S4 study) of the Michael J. Fox Foundation for Parkinson’s Research. With the current methods of aSyn detection in tissue and biological fluids, no conclusive intraindividual pattern of aSyn distribution across the fluids and tissues analyzed has been found yet, but the immunohistochemistry analysis of aSyn in the submandibular gland and skin showed promising results pointing towards a promising peripheral diagnostic biomarker even in this multicenter setting^297^.

### Gaps challenges and opportunities

Although there have been considerable efforts with building longitudinal cohorts of aSyn disorders, including prodromal conditions and improving technology, clear panels of reliably measurable and well-validated biomarkers for state, rate, fate, trait are lacking. In most biomarker studies (e.g., of CSF aSyn) there were slight differences of the mean levels in aSyn disorders vs. controls on a group level, but the high variability of single values hampers its clinical utility. Some reasons for the variability, besides blood-contamination and possibly other minor aspects of SOP^298^ could lay in the genetic background as has been recently shown with lower CSF aSyn levels in PD subjects with GBA mutations^299,300^. However, other reasons for the variability are largely unknown. Possibly, the existing clinical heterogeneity with different pheno- and progression types of PD, that is partially due to comorbidities^301^ and possibly also due to comedication and other reasons beyond the disease itself complicate the analyses. Several publications have suggested different phenotypes, but its validation is difficult due to the heterogeneity of the data captured^302^ and the statistical power even in larger cohorts like PPMI.

Therefore, additional biomarkers (e.g., NfL in blood) as a panel in the future will help to diminish this variability and overlap and hopefully also stratify different progression and subtypes of PD. With respect to cognitive decline decreased levels of β-amyloid in CSF seem to be a predictive biomarker for later cognitive decline^303^.

For clinical trials, markers of state for stratification are needed and markers of rate to objectively measure progression of the disease. So far, only dopamine transporter SPECT scans, but no biological measures, have been FDA approved to stratify PD from other diseases (like essential tremor) for clinical trials but no biological measure. Serum NfL showed an interesting increase over time, which correlated with UPDRS and some cognitive measures in one multicenter cohort^304^ but this still needs replication and validation.

The role of other modified forms of aSyn PTMs (e.g., phosphorylation) and its presence in biological fluids is still to be considered as possibly more specific biomarker based on the current literature/experience. Extensive assessment of aSyn in CSF by mass spectrometry (Hilal Lashuel, unpublished data) has failed to identify a clear pattern of PTMs of aSyn. Antibody-based ELISA techniques have reported higher levels of phosphorylated aSyn in CSF^291^. Future investigations will show if fragments of aSyn can be more specific a biomarker than the total levels.

The seeding aggregation assays show sensitivities and specificities for the PD diagnosis above 80 and 90%. A blinded comparison of two slightly different methods (RT-QuIC and PMCA) and the same CSF samples reveal high overlap^292^. The method still needs optimization to become reliably quantitative and for high-throughput measurements. The identified different seeding pattern in CSF samples of MSA vs. PD subjects suggesting different underlying strains of aSyn^70,88^, needs further validation. The challenge for the future is the improvement of this technology and its application to blood and peripheral tissues, which will be interesting for studies like the above mentioned S4 study.

Exosomes are an intriguing matrix to consider since they contain intracellular components within biological fluids. So far, exosomal aSyn has been reported in some cohorts in the CSF^305^ and also recently in a small study in plasma^306^. Still the extraction methods add variability to an anyway variable protein in biological fluids and need standardization. Once we have more specific aSyn markers, exosomal aSyn will help us to understand intracellular processes and may also lead to more accurate biomarkers in extracellular fluids.

In the future, we will also have additional biomarkers being identified by multiplex platforms, such as the proximity ligation assays, the aptamer approaches or even antibody-independent mass spectrometry techniques.

The inclusion of prodromal subjects in current PD biomarker efforts is essential. It is possible that the disease activity is much higher in the prodromal status of the disease as has been seen in Alzheimer’s disease^307^. Studying biomarkers in the most active biological time, when disease develops, is a good approach for early diagnostic biomarker for clinical trials with putative neuroprotective/-preventive strategies. These can be subjects with isolated REM sleep behavior disorder, which convert to aSyn aggregation disorders, mainly PD, on an annual rate of 7,6% according to an international multicenter study^308^ or other non-motor symptoms (hyposmia etc.) and asymptomatic mutation carriers. The Michael J. Fox Foundation for Parkinson’s Research is currently enrolling for the new PPMI cohorts: PPMI 2.0 (ClinicalTrials.gov Identifier: NCT0447778), which will be a large cohort of 4500 subjects including established PD (to study among others different pheno- and progression types) and a high number of subjects at risk to develop PD. The collected data and samples from PPMI 2.0 will be very valuable to move on with the current and future biomarker efforts.

## Outlook

*How can we turn all this aSyn research into breakthrough medicines for PD patients?*

Ultimately, the nature and extent of the aSyn-PD connection can only be determined by carefully designed clinical experiments in humans. Since the discovery of a genetic link between *SNCA* and PD^1^, there have been a large number of possible mechanisms proposed to explain this, and additional subsequent genetic links. Most are compelling and are consistent with selected aspects of PD pathology (e.g., mitochondrial dysfunction or compromised autophagy). Many have been “tested” in mouse models that recapitulate selected, but not all, features of PD^309^. Moreover, many have been “supported” by studies of *postmortem* human PD brain, which do not differentiate causes from correlates. However, none of the proposed mechanisms have been tested in PD itself. A clinical trial that measures the effect of modulating a select mechanism on disease onset and/or progression is the only way to prove/disprove any hypotheses regarding synuclein pathogenicity. It is very important, therefore, to examine the issues that are relevant to the design of a disease-modifying (as opposed to a symptom-modifying) trial of an aSyn/*SNCA*-targeted therapy. These issues fall into four interdependent categories:

1. **Patient selection:** Selection of a relatively homogeneous patient population in which aSyn/*SNCA* is a major contributor to symptom progression and/or disease onset. This patient population should progress rapidly and relatively uniformly, so that slowing of progression can be measured in a smaller, shorter trial. Identification of such a population should drive target selection. It is important to note that neither of the two recent phase 2 studies of aSyn antibodies, from Roche/Prothena and Biogen, refined the patient population except to specify disease stage (mild to moderate) and medication status (stable). The lackluster results of both studies may result directly from patient heterogeneity. In hindsight, it may have been useful to restrict patients to individuals with a specific *SNCA* snp profile or expression level. Natural history studies to test the relevance of this approach have not yet been done, but we do recommend addressing this gap in the near future to increase the probability of success for future aSyn therapeutics.
2. **Target selection:** Selection of a therapeutic strategy that is likely to produce a large and measurable effect in the chosen patient population, given that aSyn-driven pathogenesis is likely to be multifactorial. It is preferable to target upstream events like *SNCA* expression or, possibly, aSyn aggregation, as opposed to downstream effects, since any given downstream abnormality is unlikely to account for all or even most of the *SNCA* effect. It is optimal to base target selection on the genetics of disease which point directly at causality, as opposed to pathological findings (Lewy bodies, Lewy neurites, etc.), which may not be causal.
3. **Endpoint selection:** Measurement of onset and/or progression of a specific symptom that has been clearly linked to aSyn/*SNCA*. “PD” is actually a collection of often co-occurring symptoms, the progression of which can be measured in many ways. Certain genetically defined subtypes of PD are characterized by rapid cognitive loss, (GBA-PD) for example, while others spare cognition and primarily affect motor function (LRRK2-PD). Thus, it is important to determine the relationship between a measurable clinical endpoint and the targeted synuclein biochemistry.
4. **Pharmacodynamic marker selection:** To disprove any therapeutic hypothesis, it is necessary to assure that the therapeutic dose is sufficient to produce the desired biochemical effect. This requires that target engagement be measured in human brain. An example would be an imaging agent that is selective for a particular pathological form of synuclein. If a brain marker is not available, a marker in CSF, such as a specific form of aSyn associated with the disease, may suffice.

*The holy grail is a “disease-modifying” treatment for PD. So, what exactly does that mean for an aSyn drug?*

Before discussing issues of trial design in more detail, it is critical to distinguish two features of PD: age-at-onset (more precisely, age-at-diagnosis) and rate of symptom progression. A disease-modifying drug could delay PD diagnosis, slow PD symptom progression, or both. This dual effect may not be achievable, since these two phenomena may be driven by different processes, as they may be in Huntington’s disease^310^, and are in LRRK2-PD. Gain-of-kinase-function mutations in LRRK2 significantly reduce age-at-diagnosis, but lead to a form of PD that progresses relatively slowly as compared to iPD and much more slowly than GBA-PD^311,312^. Thus, LRRK2 inhibitors, which may correct the effect of the mutations, will be difficult to test in a progression trial. It is important to emphasize that mutations/polymorphisms/snps that reduce age-at-onset are more easily identified than genes that accelerate disease progression, since the latter effect requires a natural history study. One such study demonstrated that these two pools are largely separate, with only a single gene that clearly affects both: GBA1^313^. A disconnect between *SNCA* risk-associated snps and disease progression was noted previously^314^. These findings suggest that age-at-diagnosis and symptom progression could be driven by different processes, so a single therapy is unlikely to affect both, and trials must be designed accordingly.

In addition to selecting the type of disease-modifying effect that is expected, it is necessary to identify a subtype of PD where the magnitude of the *SNCA*/aSyn effect is large. These patients will be most likely to respond to an aSyn-targeted therapy. PD is not a single entity with a single cause, but multiple distinct entities with diverse causes that happen to share some symptoms (bradykinesia, tremor, stiffness, and postural instability). Other symptoms include cognitive loss, depression, REM sleep disorder, and autonomic failure. Each of these symptoms could have a different underlying cause since they do not always co-occur. The study of genetic subtypes of PD (e.g., LRRK2-PD and GBA1-PD) has made it very clear that PD subtypes exist; in the case of the subtype of PD linked to *GBA1* mutations, patients rarely develop a tremor, but and often suffer from early cognitive decline. The clinical differences between the GBA1-PD and LRRK2-PD, from each other and from “idiopathic” PD, have been detailed^315^. The fact that *LRRK2* mutations are highly penetrant but progress very slowly^312^, while GBA1-PD mutations have low penetrance and progress rapidly^316^ supports the notion that these two subtypes of PD are likely to be driven by completely different underlying biochemistries, despite the fact that a large number of studies in cell culture and animal models purport to link GBA1-PD and LRRK2-PD. Finally, it must be emphasized that a gene that is significantly associated with PD risk or progression does not mean that the magnitude of gene’s effect is large. Even though the significance of the population-wide association of *SNCA* snps with PD is clear (very low *p*-values), the effect is relatively small on a per individual basis. Genetically defined subtypes of SNCA-PD, listed below, may be an exception to this generalization and may thus be attractive therapeutics targets.

1. Very rare, autosomal-dominant familial forms of SNCA-PD are caused by missense mutations. Diverse clinical phenotypes have been reported, even within single families^317,318^. Given the scarcity of these families, it is difficult to determine the rate of progression of these forms or even whether specific genotypes (e.g., A30P, which may be an outlier) produce distinct clinical entities. For these reasons, targeting these genetic subtypes of SNCA-PD may be difficult.
2. Autosomal-dominant PD can also result from triplication or duplication of the WT *SNCA* gene, leading to the overexpression of wild-type synuclein. A second form of autosomal-dominant PD, derived from multiplication of the *SNCA* gene and overexpression of WT synuclein, has also been characterized^319^. These patients express between 200% (CNV4, triplication) and 150% (CNV3, duplication) the normal levels of WT aSyn. Triplication appears to be fully penetrant, but duplication is not - ca 50% of obligate duplication carriers identified are symptomatic^319^. These observations suggest that a modest reduction of aSyn expression could significantly reduce risk/age-at-onset. Delaying disease onset is very difficult to test clinically, and is also challenging, since dosing may have to commence before symptom onset (see below). Although there are anecdotal reports that multiplication patients progress faster than idiopathic PD patients, no published study has confirmed this, much less determined the magnitude of the difference in progression rate^319,320^. Furthermore, despite the earlier onset of CNV4-SNCA-PD, there are not large differences in progression rate of CNV4 vs. CNV3^319^. Finally, there are reports of great variability within families^317^. Together, these findings suggest that the effect of aSyn expression on the rate of PD symptom progression may be too small to justify a progression trial.
3. Large GWAS studies show that common *SNCA* snps are clearly linked to PD age-at-diagnosis, but their effects are small. Therefore, demonstration of slowed progression by a therapeutic intervention aimed at reversing the effect of the snp will require a large, long, and expensive clinical trial. Furthermore, the typical odds ratios for these *SNCA* snps are in the range of 1.3–1.4^321,322^, which means that disease in most snp-positive patients is not likely to be driven by the *SNCA* snp. One would not necessarily expect those patients to respond to a *SNCA* snp-targeting therapy. The average progression rates of patients carrying different *SNCA* snps may be distinguishable over five years of observation^323^, but executing a five-year progression trial with hundreds of patients is prohibitively expensive. It may be possible to refine a *SNCA* snp-carrying population to decrease variability in progression rate and efforts to do so must be prioritized. Using a combination of *SNCA* snps to identify fast progressing patients does not seem to be feasible^21^. It may be possible to define a PD population where progression is driven by a *SNCA* snp plus another factor. Retrospective analysis of clinical progression in a 20 patient GBA1-PD population suggests that one *SNCA* snp may affect the rate of progression of GBA1-PD, but a natural history study of a larger patient population is necessary to confirm this finding^324^. If this observation is robust, it may be possible to test a *SNCA*-directed therapy in a GBA1-PD population. This population may be hard to identify in sufficient numbers, but it will be much more homogeneous and faster progressing than the groups discussed above, so the required numbers are greatly reduced.

*Choosing an aSyn-related target that is likely to be a major driver of risk and/or progression in an identifiable subpopulation*.

1. Reducing synuclein expression. Risk-associated snps appear to affect synuclein expression in blood. If these snps correlate to aSyn expression levels in the brain, then reduction of *SNCA* translation by an antisense or RNA-directed strategy may be a viable therapeutic strategy. It is critical to be able to compare synuclein expression in brain of each *SNCA* snp and correlate with the effect on PD onset and progression. This will allow one to estimate whether it is possible to significantly modify disease progression by reducing synuclein level. In addition, it will be critical to determine whether the reduction of synuclein will have a negative consequence, especially given the wealth of data that suggests an important role for aSyn in normal biology such as in synaptic vesicle formation, docking, and release, as described above.
2. Modulating PTMs of aSyn. A significant association between PTMs of aSyn and PD age-at-diagnosis and/or progression has yet to be convincingly demonstrated. The lack of convincing evidence from GWAS studies is troubling, since one would expect that the genes encoding the enzymes that catalyze these modifications would be implicated. However, since the inhibition of such modifications is viable from a medicinal chemistry perspective, the search for a link to diagnosis/progression should be pursued.
3. Reducing a specific oligomeric form of aSyn. Many studies have attempted to link a specific oligomeric form of synuclein to PD, without success. This is very difficult to do for reasons better summarized elsewhere; one key point is that data from human brain is limited and virtually all of it comes from patients with advanced disease. Furthermore, identification and distinction of oligomeric forms is often based on antibodies that may not be selective^325^. Much of the activity around oligomeric aSyn is driven by the hope that an aSyn imaging agent, analogous to the PiB reagent that has been used for AD research, that can provide a pharmacodynamic marker for drug action in the brain. However, the AD experience (reduced image intensity does not correlate to improved cognition) should remind us that putting such an emphasis on a brain biomarker, when the significance of the species that is recognized by the imaging agent is unclear, can be counterproductive.
4. Reducing a downstream toxic effect of aSyn. This strategy is the riskiest, since it is likely that any “toxic” effect of aSyn assemblies involves more than a single pathway. Therefore, a drug that influences only one pathway will have a smaller effect size than a drug focused on several pathways.

Measuring target engagement in the brain is critical to disprove a hypothesis but is not necessary to develop a drug. Once a target and a potentially responsive subpopulation have been identified, it is optimal to develop a tool to measure the effective manipulation of that target in patients. This step is often seen as a requirement in pharma to offer the possibility of explaining a failed trial (failure to hit target of failure of idea?). However, many successful CNS drugs have been developed without proof of target engagement (Tecfidera, for example). Even worse, the focus on “biomarker-directed discovery” has led certain targets to be prioritized, despite the lack of evidence that patients suffer from the targeted abnormality (e.g., β-secretase inhibitors).

*Designing a clinical trial to detect a drug effect in delaying age-at-diagnosis, since that is the genetically validated effect of SNCA*

The possibility that PD onset and progression are driven by distinct processes is a critical consideration in the design of a clinical trial. Diagnosis of PD occurs 5-15 years after peripheral (constipation) and brainstem (REM behavior disorder) symptoms are clear, and after clear dopaminergic cell loss can be detected by imaging^290,308^. It is also becoming evident that motor and cognitive symptoms that are characteristic of PD can be detected, and their progression measured, in this prodromal phase^308^. Finally, some *SNCA* snps that are associated with age-at-PD diagnosis, are also associated with diagnosis of RBD^326^ and may affect the time-to-PD diagnosis, while other *SNCA* snps have opposite effects on RBD and PD. Further studies are necessary to enable clinical studies in prodromal PD^326^. It is also unclear when the process(es) driving diagnosis fade and those driving progression take over.

The trial design suggestions made here may not be quickly adapted, as one might tend to recruit a large number of mild-to-moderate idiopathic PD patients with the hope that large numbers can slow average progression enough to produce a low *p*-value. However, this approach comes with noticeable challenges: (1) most of the patients may not respond to the drug, (2) earlier dosing (during prodromal period) is likely to be required, and (3) the effect of synuclein on symptom progression is not yet clear. A carefully designed trial, involving RBD patients who carry one or more *SNCA* snps that are clearly linked to RBD progression focusing on delayed “conversion” from prodromal phase to PD, likely has higher chances of producing a clear signal of therapeutic benefit.

In conclusion, identifying knowledge gaps and defining research challenges and opportunities will require not only a deep understanding of what we know, but also the courage to admit what we do not know. The path forward entails: more disease natural history studies of synucleinopathies; the development of robust biomarkers; rigorous interpretation of data considering the current models’ limitations (no model recapitulates the disease but, instead, only specific aspects of aSyn pathobiology); the development of better tools/models; and last but not least, a better understanding of how to translate knowledge from research into aSyn biology into desperately needed, disease-modifying therapies for our patients.
